# Tellurite Promotes Stress Granules and Nuclear SG-Like Assembly in Response to Oxidative Stress and DNA Damage

**DOI:** 10.3389/fcell.2021.622057

**Published:** 2021-02-11

**Authors:** Aracelly Gaete-Argel, Felipe Velásquez, Chantal L. Márquez, Barbara Rojas-Araya, Constanza Bueno-Nieto, Josefina Marín-Rojas, Miguel Cuevas-Zúñiga, Ricardo Soto-Rifo, Fernando Valiente-Echeverría

**Affiliations:** ^1^Molecular and Cellular Virology Laboratory, Faculty of Medicine, Virology Program, Institute of Biomedical Sciences, Universidad de Chile, Santiago, Chile; ^2^HIV/AIDS Workgroup, Faculty of Medicine, Universidad de Chile, Santiago, Chile

**Keywords:** tellurite, DNA damage, stress granules, oxidative stress, stress response

## Abstract

Tellurium oxyanion, tellurite (*TeO*_3_^–2^), is a highly toxic compound for many organisms. Its presence in the environment has increased over the past years due to industrial manufacturing processes and has been associated with adverse effects on human health. Although tellurite induces the phosphorylation of eIF2α, DNA damage and oxidative stress, the molecular mechanisms related to the cellular responses to tellurite-induced stress are poorly understood. In this work, we evaluated the ability of tellurite to induce phosphorylation of eIF2α, stress granules (SGs) assembly and their relationship with DNA damage in U2OS cells. We demonstrate that tellurite promotes the assembly of *bona fide* cytoplasmic SGs. Unexpectedly, tellurite also induces the assembly of nuclear SGs. Interestingly, we observed that the presence of tellurite-induced nuclear SGs correlates with γH2AX foci. However, although H_2_O_2_ also induce DNA damage, no nuclear SGs were observed. Our data show that tellurite promotes the assembly of cytoplasmic and nuclear SGs in response to oxidative stress and DNA damage, revealing a new aspect of cellular stress response mediated by the assembly of nuclear stress granules.

## Introduction

Tellurium is a rare trace element found in nature and some of its derivatives, such as tellurite (*TeO*_3_^–2^) and tellurate (*TeO*_4_^–2^), induce both acute and chronic toxicity in a variety of human cells, rats and bacteria (Chasteen et al., 2009). Sustained use of this toxic agent in rubber, metallurgic and electronic industries has led to increased tellurium contamination on the environment posing a potential threat to public health (Sandoval et al., 2012). To date, most studies on tellurite toxicity have been performed on prokaryotes. A significant number of genetic tellurium resistance determinants have been identified in different species of bacteria isolated from environmental and patient samples, including bacterial species pathogenic to humans (Chasteen et al., 2009). It has been suggested that such tellurium resistance confers them a selective advantage in its natural environment (Walter and Taylor, 1992). In animals, the salts of tellurium (specially tellurite) have been found to produce severe toxic reactions, such as reversible limb paralysis due to demyelination of nerves and spinal roots (Lampert et al., 1970) as well as neurotoxic damage including learning impairment and spatial memory (Widy-Tyszkiewicz et al., 2002). Studies in human cell lines (cervical adenocarcinoma and HeLa) or murine cell lines (hepatocellular carcinoma Transplantable Liver Tumor, TLT) demonstrated that tellurite toxicity on mammalian cells is dependent on concentration and time of exposure (Ding et al., 2002; Sandoval et al., 2010). Also, it has been shown that non-transformed freshly isolated blood leukocytes are more sensitive to tellurite than human chronic myeloid leukemia cells (K562), likely due to different basal glutathione levels between both cell types (Sandoval et al., 2012). Tellurite exposure results in depletion of cellular ATP and phosphorylation of both H2AX histone and the initiation factor eIF2α, suggesting that tellurite induces DNA damage and translational arrest (Sandoval et al., 2010).

Different environmental stresses such as oxidative stress, viral infections, hypoxia, aminoacid deprivation, misfolded proteins or UV exposure activate a family of serine/threonine kinases that phosphorylate eIF2α (Kedersha et al., 2013). eIF2α phosphorylation interferes with eIF2α-GDP recycling, leading to reduced availability of eIF2/GTP/tRNA_*i*_^*Met*^ ternary complex, which suppresses translation initiation and promotes the assembly of stress granules (SGs) (Kedersha and Anderson, 2002). SGs are cytoplasmic translationally silent ribonucleoproteins described as triage centers of non-translating mRNAs. They typically contain poly(A)+ mRNAs, 40S ribosomal subunits, eIF4E, eIF4G, eIF4A, eIF4B, poly(A)-binding protein (PABP1), eIF3, eIF2, p54/Rck/DDX6, and RNA binding proteins that control mRNA stability (TIA-1, TIAR, HuR) and mRNA metabolism (G3BP-1, G3BP-2, DDX6, SMN, Staufen1, DHX36, Caprin1, ZBP1, HDAC6, ADAR). SGs have been described as signaling centers, where signaling proteins (mTOR, RACK1) and interferon-stimulated gene (ISG) products (PKR, ADAR1, RIG-I, RNase L, and OAS) accumulate (Reviewed in Gaete-Argel et al., 2019). The local concentration of signaling proteins inside SGs allows a crosstalk between multiple stress cascades, which is thought to support cell survival and determine cell fate under non-optimal conditions (reviewed in Mahboubi and Stochaj, 2017). Although SGs assembly is transient, dysregulations in their assembly/disassembly or clearance have been associated with autoimmune diseases, cancer and neurodegeneration, among others (reviewed in Mahboubi and Stochaj, 2017).

Given that tellurite induces eIF2α phosphorylation, we evaluated whether tellurite induces SGs assembly. We show the assembly of *bona fide* cytoplasmic SGs and nuclear SG-like structures in U2OS cells in response to tellurite-induced oxidative stress and DNA damage. Our observation reveals a novel aspect of tellurite cytotoxicity that is relevant in understanding its neuropathological effects previously reported.

## Materials and Methods

### Reagents and Antibodies

To induce cellular stress, sodium arsenite (arsenite, NaAsO_2_; Sigma-Aldrich) and potassium tellurite (tellurite, K_2_TeO_3_ kindly gifted by Dr. José Manuel Pérez-Donoso, UNAB, Chile) were used. Cycloheximide and Puromycin were obtained from Sigma Aldrich. The antibodies used in this work included the following: anti-phospho-eIF2α (rabbit polyclonal) and anti-GAPDH (mouse monoclonal) from Abcam; anti-eIF2α (mouse monoclonal), anti-phospho-S6K1 (rabbit monoclonal), anti-phospho-S6 (rabbit monoclonal), anti-phospho-4EBP1 (rabbit monoclonal) from Cell Signaling Technology; anti-puromycin (mouse monoclonal) from Millipore; anti-DCP1a (mouse monoclonal), anti-eIF3b (mouse monoclonal), anti-TIAR (goat polyclonal), anti-DDX3 (rabbit polyclonal), anti-phospho-mTOR (mouse monoclonal), anti-mTOR (mouse monoclonal), anti-S6K1 (mouse monoclonal), anti-S6 (mouse monoclonal), anti-4EBP1 (mouse monoclonal) from Santa Cruz Biotechnology and anti-SC35 (mouse monoclonal) kindly provided by Dr. Verónica Noches, PUC, Chile. Secondary antibodies used for immunofluorescence included the following: Alexa Fluor 594 donkey anti-mouse, Alexa Fluor 594 donkey anti-rabbit and Alexa Fluor 647 donkey anti-mouse or anti-goat from Life Technologies; and secondary antibodies for western blot (horseradish peroxidase-conjugated) anti-mouse and anti-rabbit were purchased from Jackson Immunoresearch.

### Cell Culture and Drugs Treatments

Human osteosarcoma GFP-G3BP-1 U2OS cells (Kedersha et al., 2008) were cultured and maintained in Dulbecco’s modified Eagle’s medium (DMEM, Invitrogen) supplemented with 10% fetal bovine serum and 1% antibiotics at 37°C in a 5% CO_2_ incubator. To evaluate stress granules assembly, cells were treated with 0.3 mM of arsenite for 1 h or with 0.6 mM of tellurite for 3 h unless otherwise indicated. Cycloheximide treatment was performed with 10 μg/mL for 1 h as described previously (Aulas et al., 2017). For *de novo* protein synthesis measurement, 10 μg/mL puromycin was incubated for 10 min before lysis following the protocol reported (Cinti et al., 2017).

### Immunofluorescence

After treatment, cells were washed twice in PBS 1X (phosphate buffered saline) solution, pH 7.4 (Gibco^TM^ by Life Technologies) and fixed with 4% paraformaldehyde for 20 min at room temperature. Then, cells were washed with PBS 1X, incubated with 0.1 M glycine for 10 min, washed again with PBS 1X and permeabilized with 0.2% Triton X-100 for 5 min. Finally, cells were washed 3 times with PBS 1X and stored at 4°C. For SGs markers staining, cells were blocked in 1X blocking solution (Roche) for 30 min at room temperature and subsequently, primary antibodies were applied for 1 h at 37°C. Then, cells were washed in PBS 1X followed by secondary antibodies incubation for 1 h at 37°C. After 2 washes in PBS 1X, cells were incubated with DAPI 1X for 1 min at room temperature and then washed and mounted on glass slides using Fluoromount^TM^ Aqueous Mounting Medium (Sigma-Aldrich). Confocal microscopy was performed with a Carl Zeiss LSM 700 microscope and image acquisition was carried out with a 40X objective. All imaging experiments were performed at least 2 times. Imaging analyses were performed using FiJi software (NIH).

### Live Cell Confocal Microscopy

Cells were plated on 25 mm coverslips at 70% confluency. Prior to imaging, cells were washed with PBS 1X and media was replaced with red phenol-free Tyrode medium (124 mM NaCl, 5 mM KCl, 2 mM CaCl_2_-2H_2_O, 1 mM MgCl_2_, 30 mM Glucose, 25 mM HEPES, pH7.4) supplemented with 10% fetal bovine serum and 1% antibiotics. Slides were incubated in a humidified chamber at 37°C and 5% CO_2_. Treatment with tellurite was performed at 1 mM final concentration. Images were captured every 2 min for 2 h with a 63X objective using a Carl Zeiss LSM 800 microscope and analyses were performed using Fiji Software (NIH).

### Western Blotting

Cells were collected after treatment, washed twice with PBS 1X and lysed in ice-cold lysis buffer (10 mM Tris-HCl pH 7.5, 100 mM NaCl, 0.5% NP-40, 1 mM EDTA, 0.5 mM NaVO, 10 mM NaF and protease inhibitors (Roche) adapted from Cinti et al. (2017). Protein concentration was quantified by Bradford assay (Bio-Rad) and 30 μg of lysates were denatured at 95°C in Laemmli Buffer for 10 min. Samples were resolved by SDS-PAGE and transferred to a nitrocellulose membrane, which was blocked with a 5% Blotting-Grade Blocker (Bio-Rad) and then incubated with previously mentioned primary antibodies overnight (see section “Reagents and Antibodies”). After washes with TBS-T, membranes were incubated with HRP-conjugated secondary antibodies for 1 h at room temperature and finally chemiluminescent signal was detected using Pierce^TM^ ECL Substrate. Densitometry analyses were performed using Fiji Software.

### Cell Viability Assay

For cell viability assay, cells were seeded on a 96 well plate (6 × 10^3^ cells/well) and cultured overnight. Cells were then treated with 0.6, 0.8, 1, 2 or 5 mM of tellurite for either 3, 6, or 12 h and cell viability was measured following CellTiter 96^®^ Non-Radioactive Cell Proliferation Assay (MTT) manufacturer’s instructions (Promega).

### Measurement of ROS-Induced Stress and DNA Damage

Oxidative stress detection was assessed by using CellROX^TM^ Deep Red Reagent (Thermofisher). Briefly, cells were treated with 0.6 mM tellurite for 3 h or with 50 nM H_2_O_2_ for 30 min as a positive control (Valiente-Echeverría et al., 2014). CellROX^TM^ Reagent was added at a final concentration of 1.25 μM and incubated for 30 min at 37°C. Then, medium was removed and cells were washed three times with PBS 1X, fixed with 4% paraformaldehyde for 20 min and finally mounted on glass slides using Fluoromount^TM^ Aqueous Mounting Medium (Sigma-Aldrich).

To evaluate DNA damage cells were treated with 0.6 mM tellurite for 3 h or with 50 nM H_2_O_2_ for 30 min following the experimental design described above. Then, cells were washed, fixed and subjected to indirect immunofluorescence using an anti-γH2AX antibody (Millipore). Confocal microscopy was performed with a Carl Zeiss LSM 700 microscope and image acquisition was done with a 40X objective. Imaging analyses were performed by using Fiji Software (NIH).

## Results

### Tellurite Reduces U2OS Cell Viability, Promotes eIF2α Phosphorylation and Translational Arrest

**FIGURE 1:**
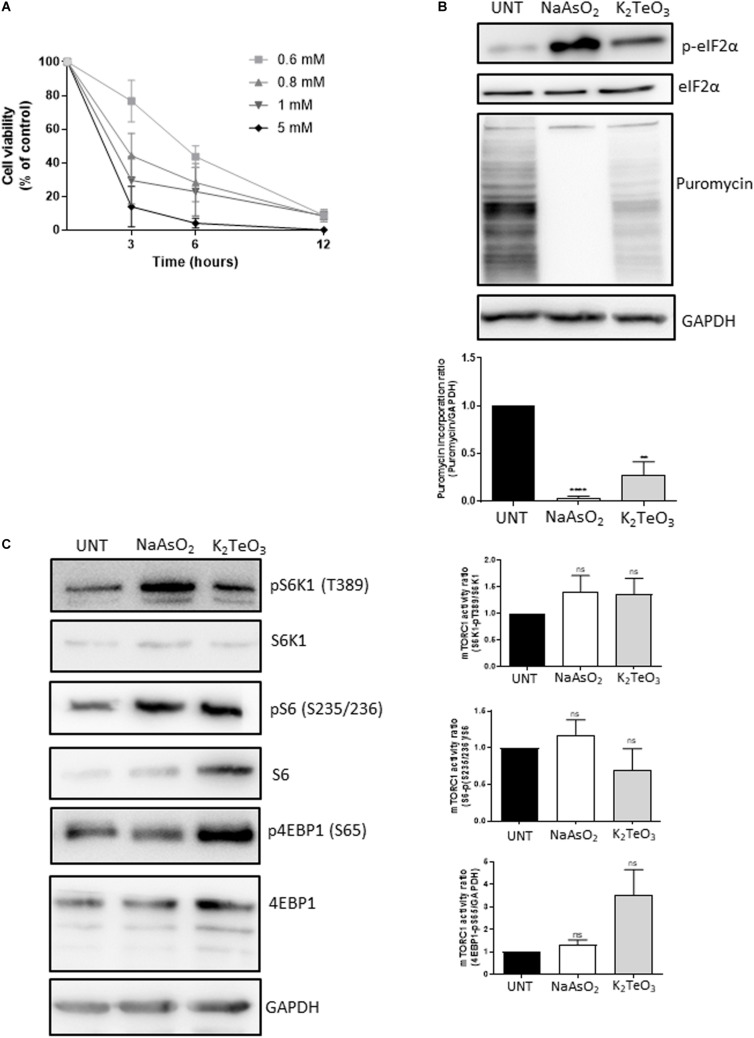
Effect of tellurite on U2OS GFP-G3BP-1 cell viability. **(A)** Cells were treated with 0.6, 0.8, 1, 2, or 5 mM tellurite (K_2_TeO_3_) for 3, 6, or 12 h. Subsequently, cell viability was evaluated using the MTT assay. Cell viability is represented as a percentage of the untreated condition. Mean ± SD from 3 independent experiments is shown. **(B)** Tellurite induces eIF2α phosphorylation and global translational arrest. U2OS GFP-G3BP-1 cells were treated with 0.6 mM tellurite (K_2_TeO_3_) for 3 h or with 0.3 mM arsenite (NaAsO_2_) for 1 h as a positive control for eIF2α phosphorylation and translational arrest. *De novo* protein synthesis was evaluated by puromycin incorporation and GAPDH was used as a loading control (upper panel). Puromycin pixel intensity was quantified by densitometry analyzes and normalized against GAPDH pixel intensity (bottom panel). Mean ± SD from 3 different is shown (****p < 0.0001, **p < 0.005 by unpaired *t*-test relative to untreated cells). **(C)** Global translational arrest is mTORC1-independent. U2OS GFP-G3BP-1 cells were treated with 0.6 mM tellurite (K_2_TeO_3_) for 3 h or with 0.3 mM arsenite (NaAsO_2_) for 1 h. The mTORC1 signaling was assesed by the phosphorylation status of its downstream targets S6K1, S6 and 4EBP1. GAPDH was evaluated as a loading control (upper panel). (Right panel) Ratio of pS6K1 (T389)/total S6K1 (upper panel), pS62 (35/236)/S6 total (middle panel) and p4EBP1 (S65)/GAPDH (bottom panel) were quantified by densitometry analyzes of figure **(C)** (bottom panel). Mean ± SD from 3 different is shown. ^*ns*^*p* > 0.05 by unpaired *t*-test relative to untreated cells.

Previous reports demonstrated that tellurite (TeO_3_^−2^) is highly toxic for most bacteria at concentrations as low as 1 mg/mL (Taylor, 1999). Compared with other metals and metalloids such as selenium, mercury, copper, cadmium, chromium and iron, tellurite toxicity occurs at 100-fold lower concentrations, demonstrating its strong effect on microorganisms (Nies, 1999). To study the toxicity of tellurite on mammalian cells, U2OS cells that stably express the stress granule (SG)-marker G3BP-1 fused to GFP (Kedersha et al., 2008) were incubated with different tellurite concentrations for up to 12 h. In previous studies where tellurite-induced toxicity was evaluated, a wide range of concentrations (1 μM–1 mM) and exposure times were used (2–24 h), showing that 1 mM tellurite induces approximately 100% death in K562 and HeLa cells at 24 h of incubation (Ding et al., 2002; Sandoval et al., 2012). Additionally, these studies revealed that tolerance to tellurite is cell-type dependent. While K562 cells retained more than 80% viability at 4 h of treatment with 0.5 mM tellurite, HeLa cells survival was less than 60% after 2 h (Ding et al., 2002l Sandoval et al., 2012). Considering this, we treated the cells with 0.6–5 mM tellurite for 3, 6, and 12 h, and cell viability was assessed. As shown in Figure 1A, cell viability significantly decreases in almost all tellurite concentrations and incubation times tested. With the exception of 0.6 mM for 3 h, all other assayed conditions reduced cell viability to less than 50% of the untreated condition, consistent with previous reports on mammalian cells (Ding et al., 2002; Sandoval et al., 2010, 2012).

It has been shown that tellurite induces eIF2α phosphorylation on the murine cancer cell line TLT (Sandoval et al., 2010). To test this, U2OS GFP-G3BP-1 cells were treated with 0.6 mM tellurite for 3 h and eIF2α status was analyzed by western blot. As a control, cells were treated with sodium arsenite, a compound known to induce the phosphorylation of eIF2α (Wheeler et al., 2016). As expected, tellurite induces phosphorylation of eIF2α (Figure 1B, upper panel), although to a lesser extent than the observed with 0.3 mM of sodium arsenite for 1 h. Since phosphorylation of eIF2α is associated to a strong decrease of protein synthesis (Gaete-Argel et al., 2019), we evaluated if tellurite influences global translation by assessing the rate of *de novo* protein synthesis through the SUnSET assay (Figure 1B, bottom panel). Notably, tellurite exposure reduces global translation as observed in the reduction of puromycin incorporation compared to untreated cells. However, its inhibitory effect is milder when compared to arsenite, which completely blocks global translation. This moderate result correlates with the lower levels of eIF2α phosphorylation obtained with tellurite compared to arsenite, suggesting that translation inhibition is related to the level of eIF2α phosphorylation.

The mammalian target of rapamycin complex 1 (mTORC1) is a key cellular regulator involved in cell growth and metabolism. Upon activation by different growth factors, nutrients or oxidative stress, it promotes protein synthesis by phosphorylation of the eIF4E-binding protein (4E-BP) and the S6 kinase 1 (S6K1), allowing translation initiation and ribosome biogenesis, respectively. Thus, to study if tellurite inhibits protein synthesis through the mTORC1 pathway, we assayed total and phosphorylated expression of S6, S6K1, and 4E-BP on cells treated with 0.6 mM tellurite for 3 h or with 0.3 mM of arsenite for 1 h. As we reported previously, arsenite-induced oxidative stress mediates the activation of mTORC1 (Cinti et al., 2017; Figure 1C). When compared to untreated cells, tellurite-treated cells exhibited enhanced phosphorylated 4EBP1 (S65) and S6K1 (pT389), indicating that tellurite enhances mTOR activity. Together, these results reveal that tellurite induces eIF2α phosphorylation and subsequently decreases global translation initiation in a mTORC1-independent manner.

### Tellurite Induces the Assembly of Cytoplasmic SGs and Nuclear SG-Like Structures

**FIGURE 2:**
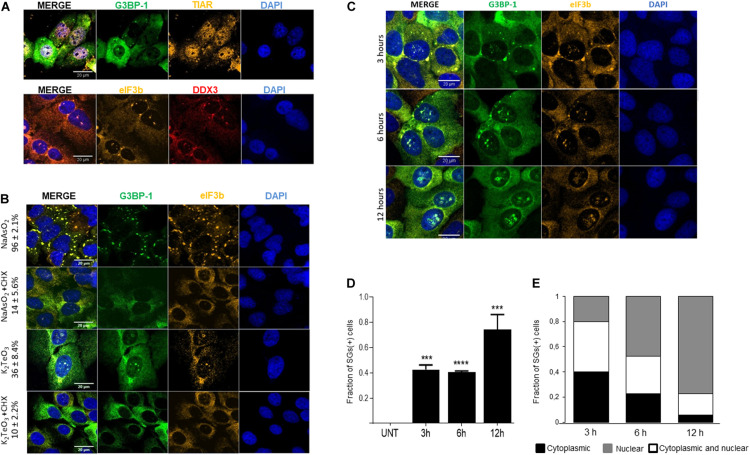
Tellurite treatment induces stress granules (SGs) assembly. **(A)** U2OS GFP-G3BP-1 cells were treated with 0.6 mM tellurite (K_2_TeO_3_) for 3 h and assessed for SG markers by indirect immunofluorescence. Tellurite-induced SGs contain GFP-G3BP-1 (green) and TIAR (yellow) as shown in the upper panel and DDX3 (red) and eIF3b (yellow) in the bottom panel. Nuclei were stained with DAPI (blue). Images are representative from 2 different experiments. **(B)** Tellurite-induced SGs are disassembled by cycloheximide (CHX). U2OS GFP-G3BP-1 cells were exposed to 0.3 mM arsenite (NaAsO_2_) or 0.6 mM tellurite (K_2_TeO_3_) for 1 and 3 h, respectively. Then, cells were co-incubated for an additional 1 h with CHX and immunostained for eIF3b (red). Nuclei were stained with DAPI (blue). Mean ± SD of SGs (+) cells in each condition is shown. Images are representative from 2 independent experiments. **(C)** Tellurite promotes cytoplasmic and nuclear SGs-like formation. U2OS GFP-G3BP-1 were treated with 0.6 mM tellurite (K_2_TeO_3_) for 3, 6, or 12 h, fixed and immunostained for eIF3b (yellow). Nuclei were stained with DAPI (blue). Images are representative from 2 different experiments. **(D)** Quantification of the fraction of SG-positive cells in the presence of 0.6 mM of tellurite (K_2_TeO_3_). Data represented as means ± SD (^∗∗∗^*p* = 0.0004, ^****^p < 0.0001 relative to untreated cells by unpaired *t*-test). **(E)** Cells were treated with 0.6 mM tellurite (K_2_TeO_3_) for 3, 6, or 12 h and quantification of subcellular location of tellurite-induced SGs was performed and plotted as fraction of cells with only cytoplasmic SGs (black), both cytoplasmic and nuclear SGs-like (white) and only nuclear SGs-like (gray). Data represent 45 cells analyzed per condition.

To determine whether tellurite induces stress granules assembly, U2OS GFP-G3BP-1 cells were incubated with 0.6 mM of tellurite for 3 h, followed by immunostaining of different SGs markers. We found that cytoplasmic granules do assemble in cells exposed to tellurite (Figure 2A). The observed aggregates contain the canonical SG markers G3BP-1 and TIAR (Figure 2A, upper panel). Additionally, other well-described SG components such as DDX3 and eIF3b were also recruited to tellurite-induced aggregations (Figure 2A, bottom panel), demonstrating that these aggregations are *bona fide* stress granules. SGs being widely considered as strictly cytoplasmic ribonucleoproteins, we were surprised to find that tellurite-treated cells appeared to have several nuclear granules containing the hallmarks of SGs. These nuclear SG-like granules were not present in arsenite-treated cells (Figure 2B) and contained eIF3b, a cytoplasmic protein (Figure 2A).

Typically, stress granules are sensitive to cycloheximide (CHX) treatment, a drug known to block polysome disassembly (Wheeler et al., 2016). To determine if tellurite-induced SGs are affected by CHX, cells treated with 0.6 mM tellurite for 3 h were then incubated with 10 μg/mL CHX for 1 h in the presence of tellurite. As shown in Figure 2B, tellurite-induced SGs are disassembled upon CHX addition, similar to what is observed with arsenite. The disassembly of pre-existing SGs occurs in both the cytoplasm and the nucleus compartments, demonstrating that tellurite-induced stress granules are dynamic entities and thus their assembly is reversible.

Considering that the effect of tellurite is time and concentration dependent (Figure 1A), we evaluated the dynamics of SGs assembly over time with different tellurite concentrations. We found that approximately 50% of the cells exhibited SGs after 3 or 6 h of 0.6 mM tellurite exposure reaching near 80% after 12 h (Figures 2C,D). Surprisingly, we observed that the localization of the tellurite-induced granules was also time-dependent. At shorter times of exposure, G3BP-1 and eIF3b-positive SGs were predominantly located in the cytoplasm (Figures 2C,E). In comparison, at longer times, 77% of the cells presented SG-like assemblies in the nucleus (Figures 2C,E). When cells are exposed to a higher tellurite concentration (0.8 mM), approximately 70% of the cells exhibit cytoplasmic SGs and nuclear SG-like aggregations at 3 h (Supplementary Figures 1A,B). Interestingly, the number of cells exhibiting SGs remains constant even after 6 or 12 h of tellurite incubation, indicating that 20–30% of cells are somehow resistant to the tellurite effect at both concentrations. In addition, a higher concentration of tellurite correlates with a higher fraction of cells in which aggregations are predominantly found in the nucleus (Supplementary Figure 1C), suggesting that the nuclear SG-like structures are maintained for a longer time compared to cytoplasmic SGs. Together, our results demonstrate a new effect derived from tellurite toxicity, that is, the induction of cytoplasmic *bona fide* SGs and novel nuclear SGs-like aggregations, to which canonical SGs markers are recruited.

**FIGURE 3:**
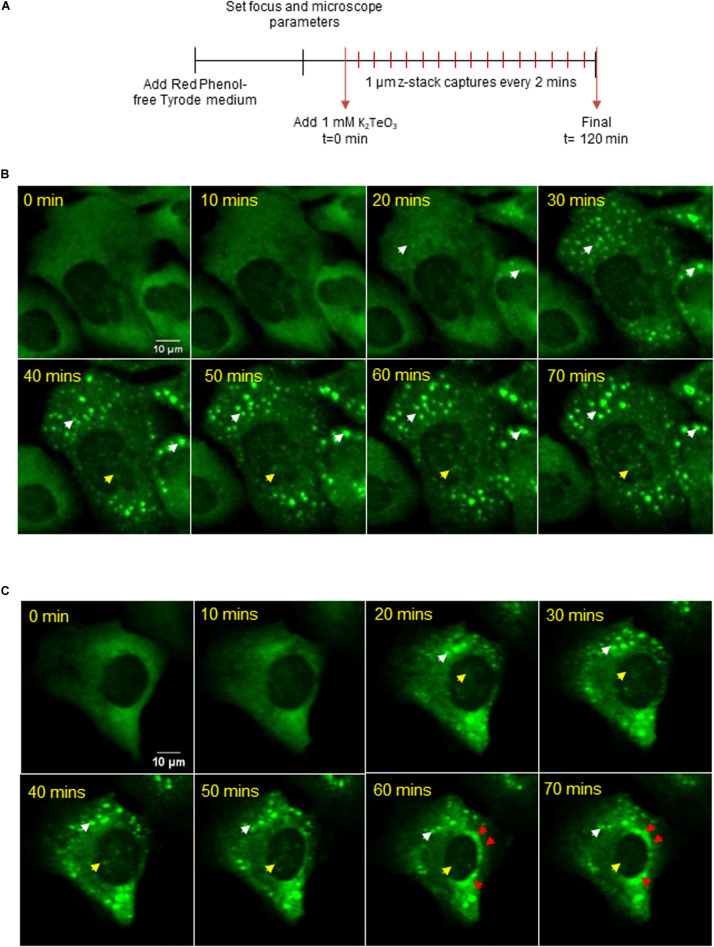
**(A)** U2OS GFP-G3BP-1 were observed by a confocal microscope under 1 mM tellurite treatment for 2 h. 12 z-stacks of 1 μm were acquired every 2 min (see Supplementary Video 1). Representative images for a 3D reconstruction of two different observed phenotypes **(B,C)** of GFP-G3BP-1 accumulation dynamics are shown (up to 70 min). White arrowheads indicate the presence of cytoplasmic GFP-G3BP-1 aggregations while yellow arrowheads indicate the assembly of GFP-G3BP-1 in the nucleus. The accumulation of GFP-G3BP-1 in a perinuclear zone is indicated by red arrowheads (bottom panel).

To determine if cytoplasmic-SGs migrate to the nucleus over time or if these SG-like structures assemble in the nucleus, we followed granule assembly using live-cell fluorescent imaging (Figure 3A and Supplementary Video 1). Given that the assembly of nuclear SG-like structures is predominant when tellurite concentration is higher (Supplementary Figures 1A,B), we treated the cells with 1 mM tellurite to observe GFP-G3BP-1 aggregations dynamics at shorter times of exposure. Microscopically visible granules can be detected approximately at 20 min after tellurite treatment in both cytoplasm (white arrowhead) and inside the nucleus (yellow arrowhead) (Figure 3B). Our observations suggest that nuclear SG-like assembly is independent of cytoplasmic SGs and can be explained as a result of G3BP-1 aggregation in both nucleus and cytoplasm (Figure 3). Additionally, we observed typical liquid-liquid phase separation (LLPS)-like properties, such as the spontaneous fusion of different aggregations and dynamical assembly/disassembly of both cytoplasmic and nuclear granules (Figure 3C, white arrowhead). Furthermore, we observed the accumulation of G3BP-1 at the perinuclear zone during tellurite exposure (Figure 3C, red arrowheads), suggesting that the aggregation of G3BP-1 inside the nucleus is a highly active process that also may requires the nuclear translocation of G3BP-1. Based on our results, we propose that tellurite induces the assembly of canonical cytoplasmic SGs as well as SG-like assemblies in the nucleus, and that over time, the cytoplasmic SGs get disaggregated by cellular machinery that is not present in the nucleus, allowing these to remain assembled in the nucleus for more extended periods.

### Tellurite Does Not Influence Other Cytoplasmic and Nuclear Membraneless Organelles

**FIGURE 4:**
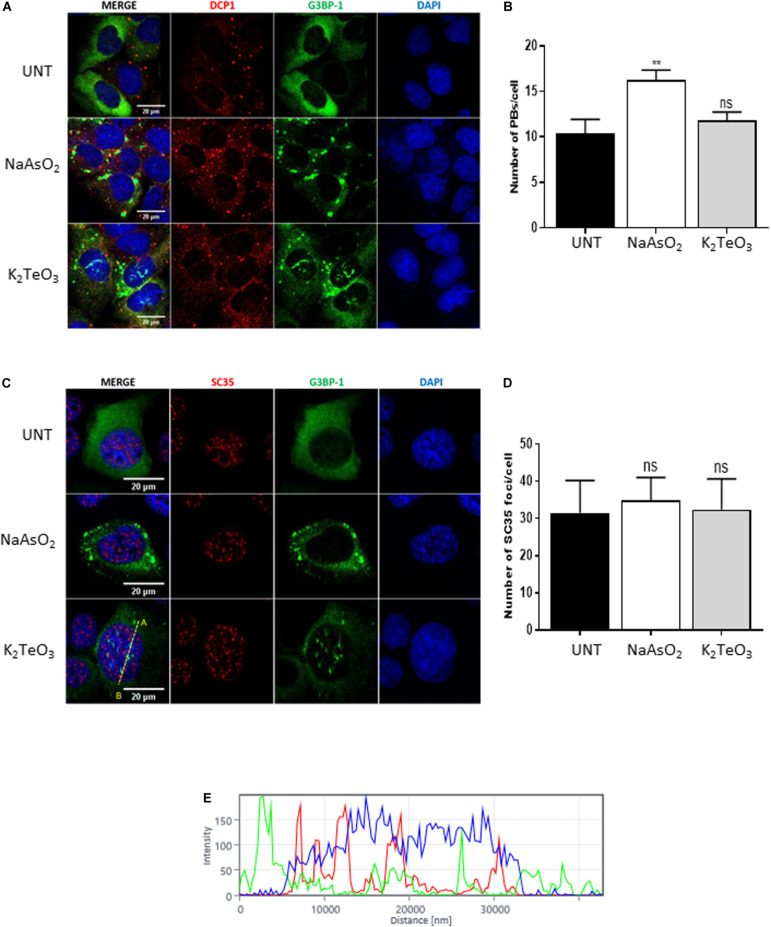
Tellurite does not affect PBs nor nuclear speckles. **(A)** U2OS GFP-G3BP-1 cells were treated with 0.3 Mm of arsenite (NaAsO_2_) for 1 h or with 0.6 mM tellurite (NaAsO_2_) for 3 h and subsequently immunostained for DCP1 (red). Nuclei were stained with DAPI (blue). **(B)** Quantification of the average PBs per cell in untreated cells or in the presence of 0.6 mM tellurite (K_2_TeO_3_) or 0.3 mM arsenite (NaAsO_2_). Data represented as means ± SD (**p < 0.01 relative to untreated cells by unpaired *t*-test). **(C)** U2OS GFP-G3BP-1 cells were treated with 0.3 Mm of arsenite (NaAsO_2_) for 1 h or with 0.6 mM tellurite (K_2_TeO_3_) for 3 h. Cells were fixed and immunostained for SC35 (red). Nuclei were stained with DAPI (blue). **(D)** Quantification of the average SC35 foci per cell in untreated cells or in the presence of 0.6 mM tellurite (K_2_TeO_3_) or 0.3 mM arsenite (NaAsO_2_). Data represented as means ± SD (**p < 0.01 relative to untreated cells by unpaired *t*-test). **(E)** Histogram of fluorescence intensity from A to B (indicated in **C**) in the nucleus.

Stress granules are closely related to processing-bodies (PBs), a type of membraneless organelles (MLOs) that are constitutively assembled in the cytoplasm (Kedersha et al., 2005). PBs are sites where untranslated mRNA and the mRNA decay machinery accumulates, and their size and number is known to be modulated by different cell stressors (Luo et al., 2018). To test if tellurite exposure modulates the PBs dynamics, we treated GFP-G3BP-1 U2OS cells with 0.6 mM tellurite for 3 h and counted the number of PBs by immunostaining of DCP1, a canonical component of PBs (Gaete-Argel et al., 2019). We observed that exposure to tellurite did not modify the number of PBs per cell as did the treatment with sodium arsenite (Figures 4A,B). Additionally, the localization of DCP1 was not affected by tellurite treatment, suggesting that tellurite induces nuclear translocation of specific SGs components without affecting processing-bodies components.

Nuclear speckles (NS) correspond to nuclear MLOs enriched in pre-mRNA splicing factors and are thought to be involved in different gene expression steps such as splicing, mRNA modification, mRNA export, among others (Galganski et al., 2017). Due to the extraordinary induction of novel SGs-like aggregations in the nucleus (Figures 2, 3), we evaluated if tellurite modulates nuclear speckles (NS) assembly. GFP-G3BP-1 U2OS cells were incubated with 0.6 mM tellurite for 3 h followed by immunostaining of SC35, a marker of nuclear speckles (Figures 4C,D). We found that tellurite exposure does not alter neither the number nor the localization of NS (Figures 4C,D). Similarly, arsenite addition has no influence on NS assembly. Besides, we observed that NS and tellurite-induced SGs-like assemblies do not colocalize (Figure 4E), revealing that tellurite induces the formation of different nuclear granules.

### Tellurite Induces Oxidative Stress and DNA Damage

**FIGURE 5:**
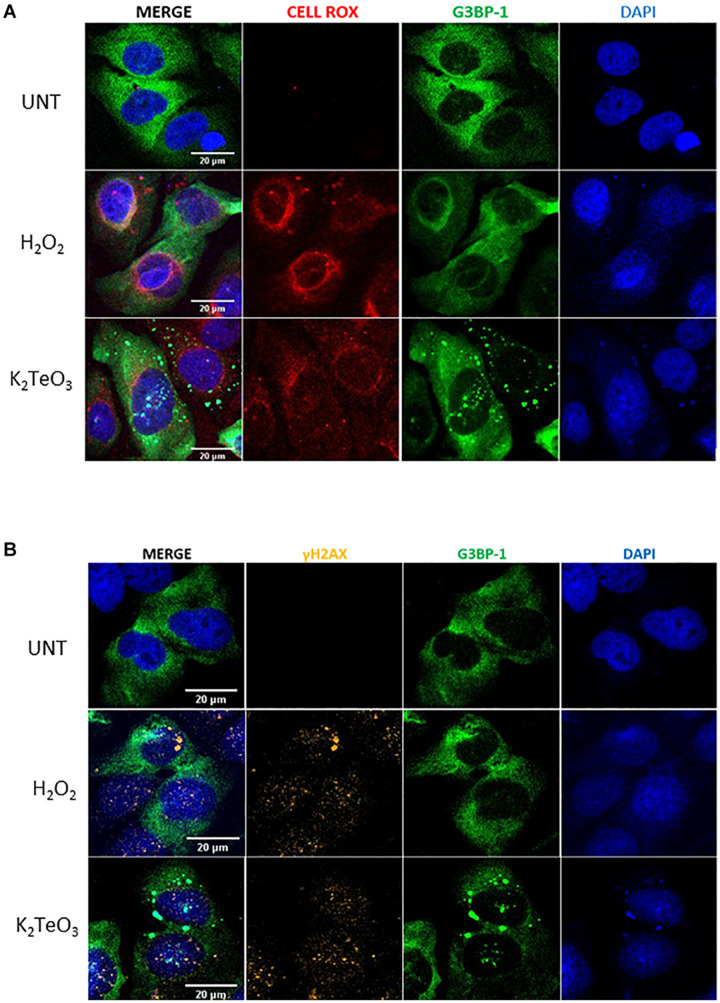
Tellurite induces oxidative stress and DNA damage. U2OS GFP-G3BP-1 cells were treated with 50 nM H_2_O_2_ for 30 min as a positive control or with 0.6 mM tellurite (K_2_TeO_3_) for 3 h followed by incubation with a fluorogenic probe (red) to detect ROS presence **(A)** or immunostained with the anti-γH2AX antibody (yellow) **(B)**. Nuclei were stained with DAPI (blue). Images are representative from 2 independent experiments.

Many studies on tellurite cytotoxicity in eukaryotic cells suggest that tellurite exposure leads to reactive oxygen species (ROS) accumulation and DNA damage (Sandoval et al., 2010, 2012). To assess whether tellurite induces oxidative stress in U2OS, cells were treated for 3 h with 0.6 mM tellurite or for 30 min with 50 nM H_2_O_2_ as positive control. After treatment, cells were incubated with a fluorogenic probe that becomes fluorescent in the presence of ROS. As shown in Figure 5A, tellurite exposure significantly induces ROS accumulation after 3 h of treatment compared to the untreated control. This result is consistent with a previous report in which different methods of ROS measurement were used (Sandoval et al., 2010). Remarkably, SGs were not observed in cells treated with 50 nM of H_2_O_2_, which is a concentration lower than the minimal 0.1 mM that is required to effectively induce SG assembly (Emara et al., 2012).

It has been demonstrated that tellurite exposure results in the presence of γ-H2AX, an early marker of double strand DNA breaks (Kuo and Yang, 2008; Sandoval et al., 2010). To study whether oxidative stress by tellurite results in DNA damage, the presence of γ-H2AX *foci* were visualized in U2OS GFP-G3BP-1 cells treated with 0.6 mM tellurite for 3 h. Figure 5B shows that tellurite exposure induces γ-H2AX accumulation, and thus DNA damage, that strongly correlates with the presence of nuclear SGs-like aggregates. However, although almost all cells treated with tellurite were positively stained for γ-H2AX, not all of them present nuclear SGs-like aggregations, probably due to insufficient G3BP-1 protein levels in the nucleus to form visible SG-like granules on those cells.

## Discussion

In this report, we demonstrated that tellurite promotes assembly of stress granules and nuclear SG-like structures in U2OS cells in response to oxidative stress and DNA damage. We showed that tellurite moderately reduces cell viability at 0.6 mM and that inhibits protein synthesis. These phenotypes correlate with increased levels of eIF2α phosphorylation while the mTORC1 pathway is active. These results are similar to the ones obtained with arsenite treatment, although we observed that arsenite induces a stronger effect. The difference between arsenite and tellurite effect could be explained by the signaling pathway occurring upstream eIF2α phosphorylation. While it has been described that arsenite activates the GNC2 and HRI kinases, a recent study suggest that BSA-tellurium nanocomposites induce eIF2α phosphorylation in a PKR-dependent manner (Zhou et al., 2018). Interestingly, the lower levels of eIF2α phosphorylation induced by tellurite could in part explain its toxic effect, as previous reports have shown that oxidative stress leads to cell death in cells expressing a mutant eIF2α (S51A) that reduces its phosphorylation (Rajesh et al., 2015).

Remarkably, we observed that tellurite induces assembly of canonical SGs as well as nuclear SG-like structures. These stress granules contain the canonical components of type-1 SGs G3BP-1, DDX3, eIF3b and TIAR (Fujimura et al., 2012). In addition, we showed that the effect of tellurite on cell viability and SGs assembly is concentration and time dependent. At more extended treatment periods, the fraction of cells presenting nuclear SGs becomes predominant, whereas cytoplasmic-SG cells become scarce. SGs are highly dynamic granules that assemble and disassemble to respond to changes in the cell environment (Buchan and Parker, 2009; Panas et al., 2016). Their clearance occurs typically after removal of the stress source, hence restoring translational activity by recruitment of SG-retained mRNAs and translation factors toward protein synthesis. However, under sustained stress, the cells often opt for removing SGs by targeting their components toward degradation as means of molecular turnover. SG removal can be aided by chaperones such as the HSP70 family and executed through autophagy, referred to as “granulophagy” (Gilks et al., 2004). In the latter, SGs are incorporated within autophagosomes and degraded in the cytoplasm (Buchan et al., 2013). Since the nucleus lacks the main components for the autophagic pathway, we propose that SGs are more easily cleared when formed in the cytoplasm than in the nucleus, resulting in the observed more permanent nuclear granules. Our results also indicate that both cytoplasmic SGs and nuclear SG-like granules are sensitive to CHX treatment, suggesting that although these nuclear mRNPs seem to be more resilient in time, they remain a dynamic entity that is disassembled in response to changes in free mRNA availability (Bounedjah et al., 2014).

Nuclear stress granule assembly has been described as a response to heat shock and, although its composition is known, their role remains enigmatic (Sandqvist and Sistonen, 2004). The previously described nuclear stress granules have been associated with sites where transcription and splicing machinery accumulates to promote a reprogramming of cell gene expression in response to heat shock (reviewed in Biamonti and Vourc’h, 2010). Here, we describe a novel type of nuclear stress granule-like structures that we hypothesize are related to the cell’s response against DNA damage induced by tellurite. Evidence regarding the role of G3BP-1 and other SG components in DNA repair support this hypothesis. G3BP-1 was identified as a poly(ADP-ribose) (pADPr)-binding protein and detected in complexes with other pADPr-binding proteins after alkylation-induced DNA damage and PARP activation mediated by Methyl-N9-nitro-N9-nitrosoguanidine (MNNG) (Isabelle et al., 2012). Additionally, it was reported that the overexpression of the poly(ADP-ribose) glycohydrolase (PARG) results in the nuclear accumulation of G3BP-1, suggesting that pADPr levels modulate the nucleocytoplasmic shuttling of G3BP-1 (Isabelle et al., 2012). Recently, the SG component FUS, but not G3BP-1 was shown to trigger the assembly of damaged DNA-enriched LLPS-like compartments in response to H_2_O_2_ (Singatulina et al., 2019). These specialized aggregations are thought to be responsible for rapid DNA repair since they concentrate DNA repair factors and reduce chromatin mobility (Wang et al., 2013; Singatulina et al., 2019). Interestingly, in none of these previously described studies, the presence of nuclear SG-like structures containing canonical components was reported. This is consistent with our observation that the exposure to H_2_O_2_ results in the accumulation of ROS and DNA damage but not in the assembly of nuclear SG-like structures. Other aspects of tellurite-induced cytotoxicity could be further explored to explain the different responses that the cell mounts against similar injuries.

As the largest eukaryotic translation initiation factor, eIF3 is a highly dynamic entity made of several subunits out of which eIF3b serves mostly as the core’s scaffold (Wagner et al., 2016). Little has been reported about the presence of eIF3 inside the nucleus, as translation is considered to occur almost exclusively in the cytoplasm (discussed in Yewdell and David, 2013). However, eIF3b does appear to have a small presence in the nucleus, along with eIF3c (Shi et al., 2009). Both eIF3f and eIF3g have been proved to shuttle to the nucleus in the context of cellular apoptosis (Shi et al., 2009; Kim et al., 2013), but no role has been established for this localization of eIF3b. In this work, we were able to observe the presence of eIF3b inside the nucleus, as part of these SG-like structures, though whether this RNA-binding protein was present in the nucleus beforehand or was imported from the cytoplasm remains unsure.

So far, stress granules assembly is a conserved pro-survival cell response to acute stress. However, the assembly of chronic SGs or defects on its clearance have been related to the progression of a wide variety of diseases, including neurodegenerative diseases (reviewed in Reineke and Neilson, 2019). Here we show that the presence and accumulation of tellurite-induced cytoplasmic, but more importantly, nuclear SG-like structures directly correlates with decreased cell metabolic activity. Also, nuclear SG-like aggregates are persistent in cells exposed to tellurite, which could be explained by the lack of autophagic machinery responsible for SG clearance in the cell nucleus. Considering these data, it would be interesting to study the possible interplay between the persistency of nuclear SG-like structures and the poorly understood neuropathological effects of tellurite.

## Data Availability Statement

The raw data supporting the conclusions of this article will be made available by the authors, without undue reservation, to any qualified researcher.

## Author Contributions

AG-A, FV-E, and RS-R designed the experiments. AG-A, FV, CM, BR-A, CB-N, and MC-Z performed the experiments. AG-A, FV, CM, BR-A, JM-R, RS-R, and FV-E wrote the manuscript. All authors listed have made a substantial, direct and intellectual contribution to the work, and approved it for publication.

## Conflict of Interest

The authors declare that the research was conducted in the absence of any commercial or financial relationships that could be construed as a potential conflict of interest.

## Supplementary Material

Supplementary Figure 1 Tellurite treatment induces cytoplasmic stress granules (SGs) and nuclear SG-like assembly. **(A)** U2OS GFP-G3BP-1 cells were treated with 0.8 mM tellurite for 3, 6 or 12 h and immunostained for eIF3b (yellow). Images are representative from 2 different experiments. **(B)** Quantification of the fraction of SG-positive cells in the presence of 0.6 mM of tellurite (K_2_TeO_3_) at 3, 6, or 12 h. Data represented as means ± SD (^****^*p* < 0.0001 relative to untreated cells by unpaired *t*-test). **(C)** Quantification of subcellular location of tellurite-induced SGs was performed and plotted as fraction of cells with only cytoplasmic SGs (black), both cytoplasmic and nuclear SGs-like (white) and only nuclear SGs-like (gray). Data represent 45 cells analyzed per condition.

Supplementary Video 1 Live cell microscopy of tellurite-induced SGs and SG-like dynamics. U2OS GFP-G3BP-1 cells were treated with 1 mM tellurite for 140 min and images were acquired every 2 min. Scale bar: 20 μM.

## References

[B1] Aulas A. Fay M. M. Szaflarski W. Kedersha N. Anderson P. Ivanov P. (2017). Methods to classify cytoplasmic foci as mammalian stress granules. *J. Vis. Exp*. 123 e55656. 10.3791/55656 PMC5607937 28570526

[B2] Biamonti G. Vourc’h C. (2010). Nuclear stress bodies. *Cold Spring Harb. Perspect. Biol*. 2:a000695. 10.1101/cshperspect.a000695 20516127 PMC2869524

[B3] Bounedjah O. Desforges B. Wu T. D. Pioche-Durieu C. Marco S. Hamon L. (2014). Free mRNA in excess upon polysome dissociation is a scaffold for protein multimerization to form stress granules. *Nucleic Acids Res*. 42 8678–8691. 10.1093/nar/gku582 25013173 PMC4117795

[B4] Buchan J. R. Parker R. (2009). Eukaryotic stress granules: the ins and outs of translation. *Mol. Cell* 36 932–941. 10.1016/j.molcel.2009.11.020 20064460 PMC2813218

[B5] Buchan J. R. Kolaitis R. M. Taylor J. P. Parker R. (2013). Eukaryotic stress granules are cleared by autophagy and Cdc48/VCP function. *Cell* 153 1461–1474. 10.1016/j.cell.2013.05.037 23791177 PMC3760148

[B6] Chasteen T. G. Fuentes D. E. Tantalean J. C. Vasquez C. C. (2009). Tellurite: history, oxidative stress, and molecular mechanisms of resistance. *FEMS Microbiol. Rev*. 33 820–832. 10.1111/j.1574-6976.2009.00177.x 19368559

[B7] Cinti A. Le Sage V. Milev M. P. Valiente-Echeverría F. Crossie C. Miron M. J. (2017). HIV-1 enhances mTORC1 activity and repositions lysosomes to the periphery by co-opting Rag GTPases. *Sci. Rep*. 7:5515. 10.1038/s41598-017-05410-0 PMC5511174 28710431

[B8] Ding W. J. Hasegawa T. Peng D. Hosaka H. Seko Y. (2002). Preliminary investigation on the cytotoxicity of tellurite to cultured HeLa cells. *J. Trace Elem. Med. Biol*. 16 99–102. 10.1016/s0946-672x(02)80035-9 12195732

[B9] Emara M. M. Fujimura K. Sciaranghella D. Ivanova V. Ivanov P. Anderson P. (2012). Hydrogen peroxide induces stress granule formation independent of eIF2α phosphorylation. *Biochem. Biophys. Res. Commun*. 423 763–769. 10.1016/j.bbrc.2012.06.033 22705549 PMC3399031

[B10] Fujimura K. Sasaki A. T. Anderson P. J. (2012). Selenite targets eIF4E-binding protein-1 to inhibit translation initiation and induce the assembly of non-canonical stress granules. *Nucleic Acids Res*. 40 8099–8110. 10.1093/nar/gks566 22718973 PMC3439927

[B11] Gaete-Argel A. Márquez C. L. Barriga G. P. Soto-Rifo R. Valiente-Echeverría F. (2019). Strategies for success. viral infections and membraneless organelles. *Front. Cell. Infect. Microbiol*. 9:336. 10.3389/fcimb.2019.00336 31681621 PMC6797609

[B12] Galganski L. Urbanek M. O. Krzyzosiak W. J. (2017). Nuclear speckles: molecular organization, biological function and role in disease. *Nucleic Acids Res*. 45 10350–10368. 10.1093/nar/gkx759 28977640 PMC5737799

[B13] Gilks N. Kedersha N. Ayodele M. Shen L. Stoecklin G. Dember L. M. (2004). Stress granule assembly is mediated by prion-like aggregation of TIA-1. *Mol. Biol. Cell* 15 5383–5398. 10.1091/mbc.e04-08-0715 15371533 PMC532018

[B14] Isabelle M. Gagné J. P. Gallouzi I. E. Poirier G. G. (2012). Quantitative proteomics and dynamic imaging reveal that G3BP-mediated stress granule assembly is poly (ADP-ribose)-dependent following exposure to MNNG-induced DNA alkylation. *J. Cell Sci*. 125 4555–4566. 10.1242/jcs.106963 22767504

[B15] Kedersha N. Anderson P. (2002). Stress granules: sites of mRNA triage that regulate mRNA stability and translatability. *Biochem. Soc. Trans*. 30 963–969. 10.1042/bst0300963 12440955

[B16] Kedersha N. Ivanov P. Anderson P. (2013). Stress granules and cell signaling: more than just a passing phase? *Trends Biochem. Sci*. 38 494–506. 10.1016/j.tibs.2013.07.004 24029419 PMC3832949

[B17] Kedersha N. Stoecklin G. Ayodele M. Yacono P. Lykke-Andersen J. Fritzler M. J. (2005). Stress granules and processing bodies are dynamically linked sites of mRNP remodeling. *J. Cell Biol*. 169 871–884. 10.1083/jcb.200502088 15967811 PMC2171635

[B18] Kedersha N. Tisdale S. Hickman T. Anderson P. (2008). Real−time and quantitative imaging of mammalian stress granules and processing bodies. *Methods Enzymol*. 448 521–552. 10.1016/s0076-6879(08)02626-8 19111193

[B19] Kim J. T. Lee S. J. Kim B. Y. Lee C. H. Yeom Y. I. Choe Y. K. (2013). Caspase-mediated cleavage and DNase activity of the translation initiation factor 3, subunit G (eIF3g). *FEBS Lett*. 587 3668–3674. 10.1016/j.febslet.2013.09.027 24080033

[B20] Kuo L. J. Yang L. X. (2008). γ-H2AX-a novel biomarker for DNA double-strand breaks. *In Vivo* 22 305–309. 18610740

[B21] Lampert P. Garro F. Pentschew A. (1970). Tellurium neuropathy. *Acta Neuropathol.* 15 308–317. 10.1007/BF00684729 5451184

[B22] Luo Y. Na Z. Slavoff S. A. (2018). P-bodies: composition, properties, and functions. *Biochemistry* 57 2424–2431. 10.1021/acs.biochem.7b01162 29381060 PMC6296482

[B23] Mahboubi H. Stochaj U. (2017). Cytoplasmic stress granules: dynamic modulators of cell signaling and disease. *Biochim. Biophys. Acta Mol. Basis Dis*. 1863 884–895. 10.1016/j.bbadis.2016.12.022 28095315

[B24] Nies D. H. (1999). Microbial heavy-metal resistance. *Appl. Microbiol. Biotechnol*. 51 730–750. 10.1007/s002530051457 10422221

[B25] Panas M. D. Ivanov P. Anderson P. J. (2016). Mechanistic insights into mammalian stress granule dynamics. *J. Cell Biol*. 215 313–323. 10.1083/jcb.201609081 27821493 PMC5100297

[B26] Rajesh K. Krishnamoorthy J. Kazimierczak U. Tenkerian C. Papadakis A. Wang S. (2015). Phosphorylation of the translation initiation factor eIF2 α at serine 51 determines the cell fate decisions of Akt in response to oxidative stress. *Cell Death Dis*. 6:e1591. 10.1038/cddis.2014.554 25590801 PMC4669752

[B27] Reineke L. C. Neilson J. R. (2019). Differences between acute and chronic stress granules, and how these differences may impact function in human disease. *Biochem. Pharmacol*. 162 123–131. 10.1016/j.bcp.2018.10.009 30326201 PMC6421087

[B28] Sandoval J. M. Leveque P. Gallez B. Vasquez C. C. Buc Calderon P. (2010). Tellurite-induced oxidative stress leads to cell death of murine hepatocarcinoma cells. *Biometals*. 23 623–632. 10.1007/s10534-010-9316-2 20213267

[B29] Sandoval J. M. Verrax J. Vásquez C. C. Calderon P. B. J. M. (2012). A comparative study of tellurite toxicity in normal and cancer cells. *Mol. Cell. Toxicol*. 8 327–334. 10.1007/s13273-012-0040-6

[B30] Sandqvist A. Sistonen L. J. (2004). Nuclear stress granules the awakening of a sleeping beauty? *J. Cell Biol*. 164 15–17. 14709538 10.1083/jcb.200311102 PMC2171964

[B31] Singatulina A. S. Hamon L. Sukhanova M. V. Desforges B. Joshi V. Bouhss A. (2019). PARP-1 activation directs FUS to DNA damage sites to form PARG-reversible compartments enriched in damaged DNA. *Cell Rep*. 27 1809.e–1821.e. 31067465 10.1016/j.celrep.2019.04.031

[B32] Shi J. Hershey J. W. Nelson M. A. (2009). Phosphorylation of the eukaryotic initiation factor 3f by cyclin-dependent kinase 11 during apoptosis. *FEBS Lett*. 583 971–977. 10.1016/j.febslet.2009.02.028 19245811 PMC2666973

[B33] Taylor D. E. (1999). Bacterial tellurite resistance. *Trends Microbiol*. 7 111–115. 10.1016/s0966-842x(99)01454-7 10203839

[B34] Valiente-Echeverría F. Melnychuk L. Vyboh K. Ajamian L. Gallouzi I. E. Bernard N. (2014). eEF2 and Ras-GAP SH3 domain-binding protein (G3BP1) modulate stress granule assembly during HIV-1 infection. *Nat Commun*. 5 1–17. 10.1038/ncomms5819 PMC4978539 25229650

[B35] Wagner S. Herrmannová A. Šikrová D. Valášek L. S. (2016). Human eIF3b and eIF3a serve as the nucleation core for the assembly of eIF3 into two interconnected modules: the yeast-like core and the octamer. *Nucleic Acids Res*. 44 10772–10788. 10.1093/nar/gkw972 27924037 PMC5159561

[B36] Walter E. G. Taylor D. E. (1992). Plasmid-mediated resistance to tellurite: expressed and cryptic. *Plasmid* 27 52–64. 10.1016/0147-619x(92)90006-v 1741460

[B37] Wang W. Y. Pan L. Su S. C. Quinn E. J. Sasaki M. Jimenez J. C. (2013). Interaction of FUS and HDAC1 regulates DNA damage response and repair in neurons. *Nat. Neurosci*. 16 1383–1391. 10.1038/nn.3514 24036913 PMC5564396

[B38] Wheeler J. R. Matheny T. Jain S. Abrisch R. Parker R. (2016). Distinct stages in stress granule assembly and disassembly. *Elife* 5:e18413. 10.7554/eLife.18413 PMC5014549 27602576

[B39] Widy-Tyszkiewicz E. Piechal A. Gajkowska B. Śmiałek M. (2002). Tellurium-induced cognitive deficits in rats are related to neuropathological changes in the central nervous system. *Toxicol. Lett*. 131 203–214. 10.1016/s0378-4274(02)00050-4 11992740

[B40] Yewdell J. W. David A. J. (2013). Nuclear translation for immunosurveillance. *Proc. Natl. Acad. Sci. U.S.A*. 110 17612–17613. 10.1073/pnas.1318259110 24143809 PMC3816431

[B41] Zhou Y. Bai Y. Liu H. Jiang X. Tong T. Fang L. (2018). Tellurium/bovine serum albumin nanocomposites inducing the formation of stress granules in a protein kinase R-dependent manner. *ACS Appl. Mater. Interfaces* 10 25241–25251. 10.1021/acsami.8b09402 29993233

