# Supplementary figures and images for: Tellurite Promotes Stress Granules and Nuclear SG-Like Assembly in Response to Oxidative Stress and DNA Damage
Source: Front Cell Dev Biol. 2021 Feb 11;9:622057. doi: 10.3389/fcell.2021.622057 (PMC7928414; doi:10.3389/fcell.2021.622057)

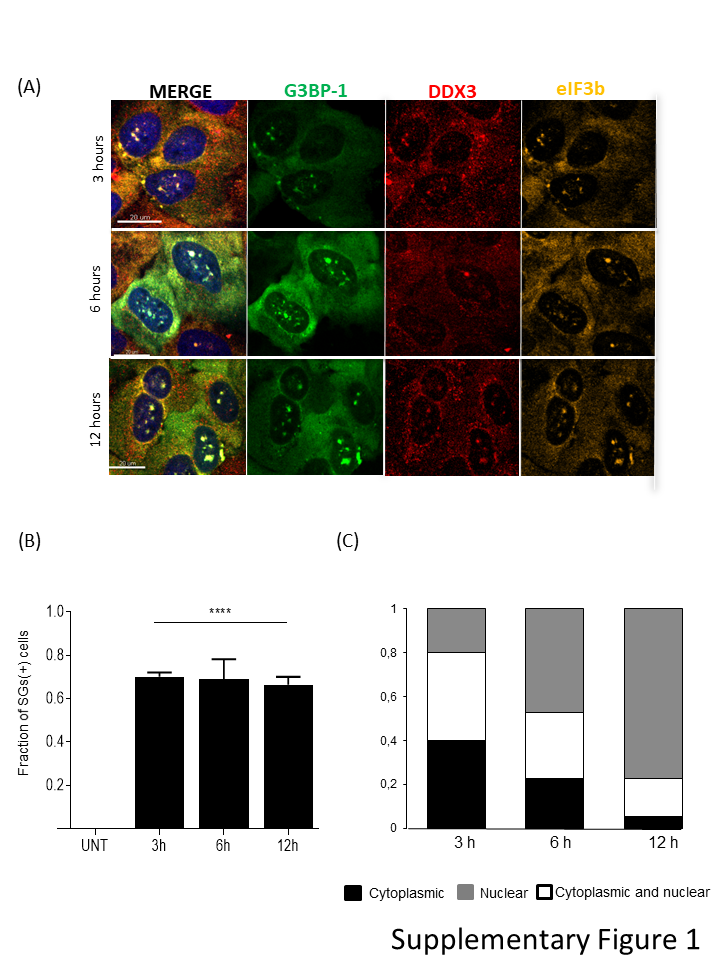

Supplement: Supplementary Figure 1 — Tellurite treatment induces cytoplasmic stress granules (SGs) and nuclear SG-like assembly. (A) U2OS GFP-G3BP-1 cells were treated with 0.8 mM tellurite for 3, 6 or 12 h and immunostained for eIF3b (yellow). Images are representative from 2 different experiments. (B) Quantification of the fraction of SG-positive cells in the presence of 0.6 mM of tellurite (K2TeO3) at 3, 6, or 12 h. Data represented as means ± SD (****p < 0.0001 relative to untreated cells by unpaired t-test). (C) Quantification of subcellular location of tellurite-induced SGs was performed and plotted as fraction of cells with only cytoplasmic SGs (black), both cytoplasmic and nuclear SGs-like (white) and only nuclear SGs-like (gray). Data represent 45 cells analyzed per condition. [file Image_1.TIF]
